# Strengthening interventions to support the sexual and reproductive health and wellbeing of young women involved in sex work amid conflict and distress migration in Ethiopia

**DOI:** 10.1080/16549716.2025.2606431

**Published:** 2026-02-04

**Authors:** Kate Pincock, Nicola Jones, Workneh Yadete, Fitsum Workneh

**Affiliations:** a Gender and Adolescence: Global Evidence (GAGE), ODI Global, London, UK; b Gender and Adolescence: Global Evidence (GAGE), Addis Ababa, Ethiopia

**Keywords:** Comprehensive sexuality education, implementation research, youth, gender, mobility, displacement

## Abstract

**Background:**

Amid growing global recognition of the importance of young people’s sexual and reproductive health and wellbeing, an intervention to support peer-based comprehensive sexuality education (CSE) for young women involved in sex work in Ethiopia was implemented in 2020. Since 2018, conflict has driven an upsurge in distress migration by young women to some intervention sites.

**Objective:**

This article explores the experiences of young women involved in sex work alongside peer facilitators to understand the strengths and limitations of the CSE intervention and the broader impacts of conflict and distress migration, in order to identify implications for future interventions in Ethiopia.

**Methods:**

The article draws on qualitative findings from implementation research undertaken with young women involved in sex work (*n* = 60) and peer facilitators (*n* = 15) in Addis Ababa, Bahir Dar and Hawassa cities.

**Results:**

Participants’ entry into sex work is characterised by an absence of supportive networks, yet their social isolation is exacerbated by the stigma of sex work. In areas affected by distress migration, adolescent girls and young women entering sex work are younger, and thus particularly vulnerable to abuse and exploitation by brokers, which further undermines their sexual and reproductive health and rights.

**Conclusion:**

CSE interventions should include adolescent girls involved in sex work who are under 18, given their heightened vulnerabilities. Interventions should engage with a broader range of stakeholders, including police, social services and legal systems, to enhance the sexual and reproductive health and rights of girls and young women, and address violence and exploitation.

## Paper Context

- **Main findings:** Stigma, poverty, conflict and a lack of supportive family networks in northern Ethiopia intersect to shape experiences of sex work, driving increasingly young and vulnerable women to enter sex work in urban areas, often through exploitative processes of brokerage and recruitment.
- **Added knowledge:** Existing work has not explored how sex work in Ethiopia is affected by conflict, particularly the recent and evolving situation in the north of the country; our work highlights how this exacerbates sexual and reproduction health and rights challenges, and the implications for interventions.
- **Global health impact for policy and action:** Future interventions must target those girls and young women who may be less visible, and/or who are under-age, and must go beyond information and access to sexual and reproductive healthcare services to offer a broader range of support, including mitigation and response to gender-based violence, legal support and social protection.

## Background

There is growing recognition globally that sexual and reproductive health is integral to the rights and wellbeing of young women and the pursuit of gender equality is in line with the Sustainable Development Agenda 2030 [1–3]. In lower- and middle-income countries such as Ethiopia, young women face significant barriers to accessing quality sexual and reproductive health services, including stigma surrounding youth sexual activity, poorly trained staff, and clinics being open at inconvenient times [4,5]. To address these challenges, Ethiopia’s 2016 National Adolescent and Youth Health Strategy focused on improving young people’s access to sexual and reproductive health information and services. As a result, the government has made particular efforts to enhance health literacy (including information on sexual and reproductive health) among adolescents and youth, to improve equitable access to adolescent and youth health services, and to improve the quality of those services [6].

However, despite growing attention to addressing these challenges for young women more generally, evidence suggests that few non-governmental organisation (NGO) programmes in Ethiopia specifically target young women involved in sex work, even though this group faces particular challenges to realising their sexual and reproductive health, rights and wellbeing. Sex work is associated with heightened exposure to gender-based violence, sexually transmitted illnesses (including HIV), unintended pregnancy, and social isolation [7–11]. Research also indicates that age is an important factor in girls’ entry into and experiences of sex work. One study found that young women who were under the age of 15 when they entered sex work were more likely to experience moderate or severe depression [10]. Studies have also found that younger sex workers in Ethiopia are at greater risk of violence than older sex workers [7,11].

In 2020, a two-year war broke out in northern Ethiopia, driving hundreds of thousands of people out of rural areas and into urban centres in search of work, though they now face insecure work conditions and economic marginalisation [12]. Although there was a cessation of hostilities in November 2022 involving the Tigray People’s Liberation Front (TPLF) and the Government of Ethiopia, violent conflict in Amhara region (of which Bahir Dar is the capital) has persisted between the ethno–nationalist Fano movement and the federal government, further exacerbating risks facing girls and young women and fuelling additional distress migration. However, the consequences of this displacement for sex work in urban centres, and the implications for efforts to support the sexual and reproductive health and rights of Ethiopian young women involved in sex work, have hitherto not been examined in research studies.

Sex workers in Ethiopia are a highly mobile population, which presents a challenge for service providers as it minimises their opportunity to provide ongoing support and services. A body of evidence suggests that rural – urban migration by young women has been an integral dimension of sex work in Ethiopia for many decades. Indeed, migration to urban areas is an important livelihood strategy for young people in rural Ethiopia, whose transitions into adulthood are complicated by decreasing work opportunities in agriculture, land shortages, and the concomitant promise of a better quality of life in cities [13,14]. Rural – urban migration is a gendered phenomenon, with various studies finding that young women make up the greatest proportion of young migrants from rural to urban areas – and the numbers appear to have increased over the past decade [15–19]. Young women in rural areas are more likely than their male peers to be unemployed or to work in the informal sector, and research finds that they move to urban areas to expand their educational and employment opportunities and escape sociocultural constraints (such as expectations of marriage and childrearing) on their futures [15,16,18,20,21].

Over the past six years, conflict in Ethiopia and its after-effects have further shifted the landscape of mobility and migration, with significant and underexplored consequences, both for sex workers and for all young people in terms of their ability to realise their sexual and reproductive health and rights. In 2020, the Internal Displacement Monitoring Centre recorded more than 1.7 million people newly displaced in Ethiopia – the highest number of any country after the Democratic Republic of the Congo and Syria [22]. This figure includes more than half a million ethnic Amhara displaced from other regions where they had been living, who came back to Amhara between 2018 and the start of 2021 [22]. Food shortages resulting from landlessness and drought have been the principal drivers of migration within Amhara to urban centres such as Bahir Dar [23]. Although cities have been attracting rural migrants for decades – in 2013, recent migrants accounted for 25% of the total population of Bahir Dar [16] – the city’s rapid expansion over the past decade places this figure at closer to 30% in more recent studies [24]. Bahir Dar’s migrant population is also overall much younger than the local population and comprised mostly women [24].

Indeed, the majority of rural – urban migrants in Ethiopia are female, with the most up-to-date, large-scale research finding that they tend to be adolescents and young adults with an average age of 22 years [16]. However, formal work opportunities for young women in urban centres are limited and competitive, and rapid growth in the Ethiopian economy has largely failed to translate into more jobs for young people. As a consequence, many young women who have migrated to urban areas in search of a better future end up employed in domestic service or sex work – often beginning in the former and transitioning into the latter due to limited opportunities and poor working conditions [25,26]. In fact, other studies have found that sex work is a consequence rather than a driver of female rural – urban migration [27]. Most studies, however, focus on internal migration to the capital (Addis Ababa) because of its significance as a destination for internal migrants [e.g. 18]. There has been less attention to the experiences of mobile young women involved in sex work in other urban centres. Although research has documented trafficking for the sex industry, there has been no coordinated policy response by the government [28].

This upsurge in movement into urban areas has also exacerbated challenges for the accessibility and uptake of sexual and reproductive health information, services and supplies in affected locales. Previous studies across Ethiopia found that young female migrants report high rates of sexual risk-taking such as not using condoms, having sex while under the influence of drugs or alcohol, and sex with multiple consecutive partners [29–31]. Forced migration and displacement also significantly increase the risk of sexual exploitation of young people who are already vulnerable [32–35].

In 2020, the United Nations Population Fund (UNFPA) launched its *International technical and programmatic guidance on out-of-school comprehensive sexuality education (CSE)*. A multi-phased intervention to expand access to CSE, linked to the UNFPA guidance and entitled ‘Reaching those most left behind through CSE for out-of-school young people’, was then implemented in Ethiopia and 11 other countries with various groups of out-of-school young people, with support from local specialist NGOs. In Ethiopia, one of the groups selected was young women involved in sex work (aged 18–26 years), given their marginalisation from sexual and reproductive health information and services, and their greater likelihood of poor sexual health and wellbeing outcomes.

The international guidance from UNFPA was adapted by UNFPA Ethiopia with input from government agencies and NGOs, leading to publication of a manual for *Sexual and reproductive health and life skills education*. The manual included topics such as contraceptive use, prevention and treatment of HIV and STIs, gender-based violence, and life skills (including communication skills, self-care, and critical thinking). It also included details of where young women could access health-related information and services and referral mechanisms. A local NGO called DKT-Ethiopia (DKT-E) was then supported to provide training sessions on sexual and reproductive health and life skills (based on the manual) to young women involved in sex work in Hawassa city (Sidama region), Bahir Dar city (Amhara region), Adama city (Oromia region) and Addis Ababa City Administration. DKT-E selected peer facilitators to run the sessions, who were trained by experts from the Family Guidance Association of Ethiopia and other NGOs working on sexual and reproductive health and rights.

Peer facilitators received an initial 8 days’ training on using the manual so as to train young women involved in sex work, and 2 days’ refresher training several months later. All peer facilitators had experience of the sex work industry, either having worked with sex workers during a previously UNFPA-funded programme (WISE-UP) or having previously undertaken sex work themselves. The former therefore had experience of delivering training and information on sexual and reproductive health and rights, and knowledge of referral systems.

Peer facilitators were trained to use interactive methods (including demonstrating how to put on condoms, videos about specific topics, moderated group discussions, and participant role play). A central objective was to create a safe environment to encourage sharing of experiences. The peer facilitators conducted a day-long session on each topic in the manual (8 days in total) in locations that were accessible to young women involved in sex work, including the offices of DKT-E and rooms in less visible locations. Trainings also started later in the day to accommodate participants’ working hours.

The CSE intervention was completed in 2020 and subsequently evaluated in 2022 through implementation research. The implementation research was funded by the World Health Organization (WHO) and carried out by the Gender and Adolescence: Global Evidence (GAGE) programme (based at ODI Global, London, UK) in collaboration with Quest Ethiopia, a local research organisation based in Addis Ababa. The overall objective of the implementation research was to assess the feasibility, acceptability and effectiveness of activities to prepare and support facilitators to deliver CSE in out-of-school settings to defined groups of young people known to face particular barriers and challenges to accessing information and services that support their sexual and reproductive health and wellbeing (see [36] for a full list of study sub-objectives). A data analysis manual prepared by WHO was used to guide initial coding of transcribed and translated qualitative interviews and focus group discussions. It linked different qualitative research questions used in the qualitative toolkit with the different study sub-objectives, with codes assigned to each question [36].

However, it did not attend to the conflict-related impacts on young women involved in sex work. Nonetheless, because of the implementation of the CSE intervention during the conflict period in Ethiopia, conflict emerged as a secondary theme for further attention and analysis. This further round of analysis enabled the researcher to address key evidence gaps in the extant literature as to the consequences of social and political upheaval and rural displacement for sex work in urban centres in Ethiopia, and the implications for efforts to support the sexual and reproductive health and rights of young women involved in sex work. This article therefore draws on this data to explore the impacts of the recent conflict and related distress migration on the dynamics of sex work in Ethiopia from the perspectives of various stakeholders, including young women, and its implications for future interventions.

## Methods

### Study design

To examine the impact of conflict and distress migration on sex work in Ethiopia and identify the consequences for CSE interventions for young women, we undertook further analysis of the data generated through the implementation research study with three groups of participants.

The first group were young women involved in sex work in Addis Ababa, Bahir Dar and Hawassa. Alongside questions about their experiences of the CSE intervention specifically, they were also invited to talk about how they became involved in sex work. Young women involved in different types of sex work (street-based, hotel-based, house-based) were purposively sampled to explore both how young women enter the industry, and how the type of sex work they engage in affects their exposure to risk, level of knowledge, access to support, and other factors relating to sexual health and wellbeing. During data collection it became clear that it was difficult to categorise young women involved in sex work according to place of work, because their locations are not fixed and often change over time. Young women who participated in the interviews and focus group discussions were aged between 18 and 26 years; the project itself only included young women who were aged 18 or over and this was the cohort that the research sample was drawn from.

The second group were peer facilitators. During interviews and focus group discussions, we explored (through open questioning) their perceptions of the challenges encountered by young women involved in sex work, and the consequences for their sexual health and overall wellbeing. Discussions also explored peer facilitators’ experiences of the instruction and support they received for delivering the training sessions, and their reflections on the intervention’s outcomes for participants.

The third group were key informants. We undertook interviews with project management staff in each location to explore their perceptions of the key challenges facing young women involved in sex work, and the impacts of those challenges on their sexual health and wellbeing.

### Data collection

Data collection was initially planned for 2020 and 2021, but due to the Covid-19 pandemic, both the project launch and the implementation research were delayed, with research eventually completed between July and August 2022. Due to time and budgetary constraints, data was collected in three of the four implementation sites (Addis Ababa, Bahir Dar and Hawassa). Data was collected by experienced researchers using qualitative and quantitative tools (see Table 1 for the research sample and the Appendix for a list of the research tools). For the interviews with young women involved in sex work, GAGE recruited female researchers who had several years’ experience collecting mixed-methods data on sensitive topics related to gender, adolescence, youth, disability and sexuality, and who had knowledge of the context in each study site. Researchers all had at least a second degree. Interviews were undertaken in settings identified by DKT-E project staff as safe, appropriate and accessible in each location, which was often the NGO’s offices or drop-in centres that young women involved in sex work were familiar and comfortable with.

**Table 1.** Research sample.

Research site	Individual interviews with sex workers	Individual interviews with peer facilitators	Key informant interviews	Focus group discussions with sex workers	Focus group discussions with peer facilitators	
Addis Ababa	8	3	2	2 (1 per site, 6 participants in each)	1 per site, 2 participants	
Bahir Dar	8	3	2	2 (1 per site, 6 participants in each)	1 per site, 2 participants	
Hawassa	8	3	1	2 (1 per site, 6 participants in each)	1 per site, 2 participants	
**Total number of participants**	**24**	**9**	**5**	**36**	**6**	**80**

### Data analysis

An inductive approach was identified as the most appropriate analytic strategy because of the absence of existing work on or theories about the connections between conflict, mobility, sex work, and sexual and reproductive health and rights in urban Ethiopia [see 37,38]. The inductive analysis focused on identifying the drivers and consequences of young women’s involvement in sex work in the intervention sites and at the moment in time when the research was undertaken, which was characterised by widespread conflict and distress migration, especially affecting young women in Bahir Dar. As they read through transcripts, coders assigned additional codes that were derived inductively from the data, in addition to the deductive analysis that was undertaken based on the implementation research analytical framework. Analysis was refined through discussion between research team members about the assigned codes and interpretation of data until consensus had been reached. All coding was undertaken using the software MAXQDA.

### Ethical considerations

Research ethics approval was obtained locally from the Ethiopian Society of Sociologists, Social Workers and Anthropologists. The tools were translated into Amharic for use in all field sites. Tools, information sheets and consent/assent forms were printed and distributed to the research team, who had received training on the research project aims and use of the tools, with follow-up training on research ethics, data collection methods and sampling, and data management. The team obtained letters of support and approval from the relevant government bodies to get access to the research sites and to interview participants. They also undertook extensive communication with UNFPA and DKT-E to get access to information about the project participants and facilitators.

## Results

### Drivers and consequences of involvement in sex work

#### Social exclusion and stigma shape entry into and experiences of sex work

Many young women in Bahir Dar and Hawassa reported moving to urban centres and eventually entering sex work due to an absence of familial support networks within their home community. One young woman explained how she moved in with her grandmother when her father died, but due to her grandmother’s poverty and disability, she had to support her. When her grandmother died, she returned to her mother’s house, where she faced similar conditions:

My father died when I was very young, I was raised by my grandmother. I went into sex work after the death of my grandmother. My grandmother was blind, she was a beggar, and I was helping her. I went to school late because I was supporting her. After the death of my grandmother I started to live with my mother. My mother is poor, she has many children. I decided to sell sex and assist my mother with childcare. (Young woman involved in sex work in Bahir Dar)

Young women often described the decision to migrate as being linked to the death of a family member and the consequent impact on their informal networks of support. One young woman in Bahir Dar explained that she had moved to an urban area after her father died to look for work she could do alongside schooling. She found it increasingly challenging to attend school due to long working hours at night and, in the absence of access to social protection, decided to prioritise earning money:

I left the rural area after the death of my father. When I left … I was thinking to become a house worker. I thought no one moved to town to become a sex worker. I left my rural area at grade 6, I learnt up to grade 9 after I came here. I was attending school in night and day shifts. Last year I dropped out from grade 9 because it was difficult to continue in a day shift doing sex work at night. I was sleepy in school … I decided to focus on money. I dropped out of school and continued in sex work. I want to work, save money and change my life. (Young woman involved in sex work in Bahir Dar)

Another participant in Hawassa, explaining why she dropped out of school and entered sex work, described the death of family members and the poverty she subsequently encountered when she and the father of her children later split up:

My family all died and my brother and I become orphaned … I was not on good terms with my husband and I was having difficulty to provide for my children. (Young woman involved in sex work in Hawassa)

Young women also described social isolation linked to the stigma of sex work, and the barriers this presents for integration into local communities. One young woman in Addis Ababa explained why she hides her work from those around her:

Some people consider sex workers as vulgar and with very bad behaviour, they consider us dirty. They imagine us as thieves, vulgar and bad in general. They consider us individuals that should not socialise with the general community. No one in our area knows I am in sex work, I go in the morning and come back home in the evening, they know I am a waitress. The community does not want us to participate … They criticise sex workers and we are afraid to socialise with the community. (Young woman involved in sex work in Addis Ababa)

#### Exploitation and abuse by brokers and hotel owners

Facilitators in all project locations described a system in which hotel owners and brokers collude to exploit young women and adolescent girls, especially younger girls, who do not understand how the industry runs and are thus extremely vulnerable to being taken advantage of and badly treated, as well as becoming indebted to brokers. Facilitators in Bahir Dar also described young women as not properly understanding the nature of sex work, and thus being unable to make an informed decision about working in the industry. One facilitator explained:

The owner of a [hotel] business needs to pay for the broker, they are there to work for her/him, that’s why they pay. The owners of the hotel get paid from condom sales, room rent, selling alcohol, and they benefit from the presence of sex workers. [The sex worker] eats food by credit, she borrows money and she is under credit. (DKT-E facilitator in Bahir Dar)

One young woman described financial penalties being used by hotel owners if the young woman refuses sex – something that would not apply in street-based or home-based sex work:

In hotels, they [clients] give money first and if she refuses, he can ask her to return the money. If the guy leaves with the money, then she will also have a penalty from the hotel. If she was working on the street or at home she can go without the money, but if she works in a hotel then she will have to pay 1,000 birr. (Young woman involved in sex work in Hawassa)

Another facilitator in Addis Ababa explained how businesses exploit young women financially, relying on their lack of knowledge and a severely unequal power relationship in which young women involved in sex work have little leverage with which to negotiate:

Most young girls go to an Areqe house [venue selling local alcohol] directly from the broker. [A girl] is employed as a cashier in an Areqe house, then she goes into sex work. The owner of that business needs to demonstrate condom use to girls that start sex work, but some of them do not teach them to use condoms. Some of them take money from a sex worker and tell her ‘I will pay you a salary’. The young girls that you interviewed were giving money from sex work to the lady that was the owner of the business. (DKT-E facilitator in Addis Ababa)

In Bahir Dar, peer facilitators who were involved in delivering the intervention observed growing numbers of young women entering sex work and their visibility on the streets:

Now in the city, there are many young girls selling sex on the street. They are too many and you may be shocked if you travel on the street during the nights. In the past 10 years, we observed an increasing participation of large numbers of young women involved in sex work and they are spread out on the street searching for men’s custom. (DKT-E facilitator in Bahir Dar)

Although young women who participated in the research did not directly describe conflict-induced migration being part of their experience of entering sex work, facilitators in Bahir Dar observed a link between instability in the region and growing numbers of young women entering sex work in recent years:

The most common reason for increased numbers of young girls in sex work is related with the war, and displacement as a result of the war. Displaced young women meet brokers that work at bus stations and some of those girls are virgins when they meet brokers. Brokers rape them and tell them to go for sex work. (DKT-E facilitator in Bahir Dar)

Reflecting the growing numbers of younger women involved in sex work in the city, facilitators in Bahir Dar also described how the exploitation and abuse of younger girls by brokers has become more extreme in recent years. One facilitator explained that the lack of social networks for many young women means they are particularly vulnerable to being taken advantage of because they have no support when they arrive in the city:

They come to the cities in search of jobs and to support their families. They do not know the challenges in the urban areas. Most of them are coming to be employed as domestic workers. They do not know about sex work. They may not have any connection with any relatives or friends. They arrive in the bus station and the brokers pick them up, telling them that they can help them to be employed as domestic workers or to transit to another city. Then the brokers take them to their temporary room. Most of them may not even have had sex before and they are exposed to brutal rape. Once they are picked up from the bus station, they are put under the full control of the brokers. Then they face rape in the first day of arriving in the city. Since the brokers do not use condoms, they are HIV positive, and the girls easily face infection. (DKT-E facilitator in Bahir Dar)

### Experiences of the comprehensive sexuality education intervention

#### Positive experiences of the intervention

The young women involved in sex work who participated in the CSE training sessions generally spoke very positively about its outcomes. They described the information shared by facilitators as useful and relevant, with some stating they developed more confidence to negotiate with customers over condom use, and being less fearful of testing regularly for HIV and other STIs.

We learnt about … how to negotiate on condom use. We convince customers, telling them condoms are useful to protect both of us. I tell him, I am a sex worker and having unprotected sex with me is risky for him, he may contract disease. (Young woman involved in sex work in Addis Ababa)

Facilitators were trained to connect young women involved in sex work with sexual and reproductive health service facilities, and to advise them of their rights to access healthcare and be treated with dignity and respect by service providers:

I helped many sex workers, especially newly entered sex workers, get health services. Even, I took many sex workers to the health station and also to the Family Guidance Association to get services. (DKT-E facilitator in Hawassa)

Young women involved with sex work described being able to access health services as part of the training in Addis Ababa, and going to health facilities themselves:

I visited the health facility to test for HIV. They were happy to test me when I told them I came to test for HIV. During the training they taught us well about the test. We learnt the importance of testing, then they encourage us to test, and we all tested at the training places. (Young woman involved in sex work in Addis Ababa)

#### Limitations of the intervention for addressing broader challenges

Despite positive feedback about the intervention, the accounts of young women involved in sex work and the peer facilitators indicated that there was no engagement with stakeholders other than healthcare providers during the course of the intervention – an omission that had significant implications for the young women involved. Facilitators explained that the police and legal officials are simply not interested in protecting young women who are involved in sex work, and this makes it difficult to prevent these young women being exposed to risks:

All responsible bodies do not work towards the end of the objective of their organisation. There are police, health professionals, justice offices, and the license is given to the brokers by the Department of Social Affairs, etc. However, they are not committed to protecting the rights of women and girls. For example, the police are responsible for protecting the sex workers from any abuse on the street. We always try to connect the brokers and bar owners with the police. However, practically, they have not committed to work with these people and provide support to these vulnerable young women and girls. (DKT-E facilitator in Bahir Dar)

Facilitators and young women involved in sex work described their encounters with police in generally negative terms. Many described entrenched discrimination that left them feeling hopeless that they will see justice for violence perpetrated by clients, as one young woman explained:

[A sex worker] may report it to police and police criticise her for engaging in sex work and he tells her that it is appropriate for you, the person did well for beating you. Because of the criticism from the police, we are not motivated to report the case. We tolerate the beating and wait at home till we recover, then we go back to work after we have recovered. (Young woman involved in sex work in Bahir Dar)

Facilitators also expressed that brokers were a key obstacle to protecting the rights of young women involved in sex work, despite the intervention’s efforts to engage with them as stakeholders in the sex industry:

The brokers always say they have the license to connect women with hotel owners and domestic work employees, etc. However, we know that each of the brokers has a temporary room where they keep the newly arrived girls for some days and train them on how to engage in the sex industry. The brokers are not interested to take training on the rights of young women and girls as well as on sexual and reproductive health services and rights. (DKT-E facilitator in Bahir Dar)

## Discussion

Overall, young women involved in sex work, as well as peer facilitators with previous knowledge of working in the industry or even with direct experience of sex work themselves, were positive about the CSE intervention. Young women were especially positive about the information they received on how to protect themselves from STIs and how to access health services. However, during in-depth interviews and group discussions, interviewees raised a number of issues that impacted on the extent to which young women involved in sex work can realise their rights to sexual and reproductive health and wellbeing, even when provided with knowledge and linkages to health services. These issues underscore the critical importance of ensuring that any CSE intervention is sufficiently contextualised and tailored to local realities.

Reflecting findings elsewhere in the literature on the links between social exclusion and early sexual debut [39], young women involved in sex work across all three cities (Addis Ababa, Bahir Dar and Hawassa) described experiences of social isolation both in relation to their entry into sex work, and as a result of working in the industry. Social isolation and stigma have consequences for young women’s experiences of sex work and access to services and support. A lack of supportive social networks and distress migration leave young women highly vulnerable to exploitation and abuse by brokers.

The role played by brokers in the sex work industry in Ethiopia is debated within the extant literature. Some research finds that girls are approached and actively forced into sex work from their hometowns, whereas other research argues that such coercion is less prevalent than this discourse implies [15,18,40]. Within the context of our research, however, brokers were most often described as people who led girls into sex work *once* the girls and young women had already reached the city, and who preyed on their social isolation. Being kept isolated within a hotel also meant that girls were unable to access networks, care and services that could support their sexual and reproductive rights and wellbeing. However, these dynamics were not addressed by the CSE intervention, despite it targeting sex workers who work on the street and in hotels and brothels.

Our findings also indicate that in Bahir Dar and Hawassa, sex work undertaken in hotels or brothels is often connected to exploitative practices both by brokers and establishment owners, who take advantage of the lack of knowledge of younger women and adolescent girls who have recently arrived in an urban area. Often, street-based sex work in Ethiopia is perceived as more dangerous for young women, with sex work undertaken in hotels or brothels offering a higher degree of protection from violence by clients [41]. However, our findings suggest that hotels and brothels present particular dangers because not only are young women’s movements restricted and their earnings siphoned off, but they are unable to access sexual and reproductive health services such as HIV testing or medication. These vulnerabilities notwithstanding, the intervention did not seek to engage with brokers (though individual peer facilitators had tried to do so as part of outreach work during the previous WISE-UP programme) and as such was not able to address this exploitative power dynamic.

Indeed, when it came to other stakeholders, the intervention focused solely on linkages between young women involved in sex work and the aforementioned services. This reflects a wider focus of government health policy actors on disease prevention, which overlooks the myriad intersecting forms of stigma and social exclusion faced by sex workers that contribute to their poor sexual and reproductive health and wellbeing outcomes [42,43,44]. The intervention’s focus on healthcare demonstrated limited recognition of the need to also strengthen linkages to justice systems that could address the financial and other forms of abuse perpetrated by brokers and hotel owners. Such linkages would help to ensure that young women involved in sex work are not subject to further discrimination, abuse and stigma when seeking accountability and claiming their rights. As some young women and peer facilitators reported during our research, the police themselves often perpetrate abuse against young women involved in sex work, or do little to investigate crimes against them. This deters the young women from reporting violence or abuse.

Conflict in Amhara region has led to unprecedented rates of distress migration in parts of the country, with consequences for the profile of young women involved in sex work. In Bahir Dar especially, peer facilitators observed the growing number of much younger women entering the industry, noting that they are particularly vulnerable due to their social isolation in urban centres and their desperate need to find work and safety. However, the intervention’s inclusion criteria stipulated that only young women over the age of 18 would be eligible. This meant that it did not include young women who were in the most vulnerable and exploitative situations, and therefore at high risk of poor sexual and reproductive health knowledge, practices, outcomes, and access to services.

## Strengths and limitations

The credibility of this study is enhanced by researchers’ familiarity with the locales where the research was undertaken, their overall experience of undertaking sensitive research and engagement with reflexive principles, and the use of interviews and focus group discussions with multiple stakeholders. However, although the wider CSE evaluation research used mixed methods, these findings draw solely on qualitative research, and use small sample sizes. The findings are not transferable to other contexts, but the description of the research sample enables insights into possibilities for replication of the study. The dependability of the findings is supported by the transparency of data collection and analysis processes documented in the Methods section. Researchers also undertook peer debriefing and dissemination with participants to cross-check analytical insights and to improve confirmability of findings.

## Conclusion and recommendations

By exploring the experiences of young women involved in sex work and of peer facilitators in the urban sites where the CSE intervention was implemented, this article has shown how broader structural conditions of stigma, poverty and conflict shape experiences of sex work. Addressing these broader factors is key to strengthening interventions designed to improve sexual and reproductive health and wellbeing outcomes for young women involved in sex work.

Given the challenges that participants described within the wider social environment, it is clear that future CSE training must establish broader service linkages, especially to gender-based violence prevention and response services, as well as to social care and social protection (see below). Interventions must also work with the police, and legal and justice systems, to help create the wider scaffolding for young women’s rights to sexual and reproductive health and wellbeing.

In order to reach health services and have options to engage in alternative livelihoods or skills building, young women involved in sex work would need to have their basic living costs covered, yet they have not been supported to access social protection systems such as the Urban Productive Safety Net programme (UPSNP) that target the most vulnerable households. This is largely because the programme did not reach out to youth-headed households, and beneficiary lists were not regularly updated to cater for new urban migrants entering sex work. Stigma and abuse within the police system must also be addressed so that young women involved in sex work feel safe and can seek justice when they report violence and other crimes.

Given the key role of brokers in the recruitment of under-age girls into sex work, and also in perpetuating abuse of sex workers, they must be properly investigated and prosecuted, and officials within the justice system must receive training on the rights of sex workers. This kind of training on duty of care and accountability should also include hotel and bar owners, focusing on how they can properly protect young women’s rights whenever they are violated by clients.

As the research indicates a troubling rise in the numbers of adolescent girls and young women aged under 18 who are affected by distress migration becoming involved in sex work in Bahir Dar, as well as their exploitation and even imprisonment by brokers, it is essential that future interventions target those girls and young women who may be less visible, and/or who are under-age. Social workers should be assigned to provide proper counselling and psychosocial support to young women involved in sex work who have experienced gender-based violence, with particular attention to (and specialised support for) under-18s. To support this, social work cadres employed through the Ministry of Women and Social Affairs, as well as NGOs working on sexual and reproductive health issues, should be involved in future collaborations.

More broadly, in order to prevent distress migration – which our findings reveal is a major contributor to involvement in the sex industry – there is an urgent need to mitigate its drivers by scaling up shock-responsive social protection, including expanding the country’s flagship PSNP in both rural and urban areas, and ensuring that it targets youth-headed households. For adolescent girls and young women who do migrate, there must be efforts to ensure safe migration, including advertising services and support for young girls when they first arrive at bus stations in urban centres. Combined, these efforts would help girls and young women understand their rights to sexual and reproductive health and wellbeing, including their rights to gender justice and accountability.

## Appendix. Research tools

**Table:**

	Activity
Tool 1	Workplan and curricula review
Tool 2	Project report review
Tool 3	Key informant interviews with project management
Tool 4	In-depth interviews with facilitators
Tool 5	Focus group discussions with facilitators
Tool 6	Pre/post training assessment
Tool 7	Observation of sessions
Tool 8	In-depth interviews with young people
Tool 9	Focus group discussions with young people
